# Photopolymerization-based synthesis of iron oxide nanoparticle embedded PNIPAM nanogels for biomedical applications

**DOI:** 10.1080/10717544.2017.1373164

**Published:** 2017-09-14

**Authors:** Daniel J. Denmark, Robert H. Hyde, Charlotte Gladney, Manh-Huong Phan, Kirpal S. Bisht, Hariharan Srikanth, Pritish Mukherjee, Sarath Witanachchi

**Affiliations:** a Department of Physics, University of South Florida, Tampa, FL, USA;; b Department of Physics, University of Alabama, Tuscaloosa, AL, USA;; c Department of Chemistry, University of South Florida, Tampa, FL, USA

**Keywords:** Magnetic nanoparticles, stimuli–responsive polymer, targeted biotherapeutic delivery, induction heating, photopolymerization

## Abstract

Conventional therapeutic techniques treat patients by delivering biotherapeutics to the entire body. With targeted delivery, biotherapeutics are transported to the afflicted tissue reducing exposure to healthy tissue. Targeted delivery devices are minimally composed of a stimuli responsive polymer allowing triggered release and magnetic nanoparticles enabling targeting as well as alternating magnetic field (AMF) heating. Although more traditional methods, like emulsion polymerization, have been used to realize such devices, the synthesis is problematic. For example, surfactants preventing agglomeration must be removed from the product increasing time and cost. Ultraviolet (UV) photopolymerization is more efficient and ensures safety by using biocompatible substances. Reactants selected for nanogel fabrication were *N*-isopropylacrylamide (monomer), methylene bis-acrylamide (crosslinker), and Irgacure 2959 (photoinitiator). The 10 nm superparamagnetic nanoparticles for encapsulation were composed of iron oxide. Herein, a low-cost, scalable, and rapid, custom-built UV photoreactor with *in situ*, spectroscopic monitoring system is used to observe synthesis. This method also allows *in situ* encapsulation of the magnetic nanoparticles simplifying the process. Nanogel characterization, performed by transmission electron microscopy, reveals size-tunable nanogel spheres between 40 and 800 nm in diameter. Samples of nanogels encapsulating magnetic nanoparticles were subjected to an AMF and temperature increase was observed indicating triggered release is possible. Results presented here will have a wide range of applications in medical sciences like oncology, gene delivery, cardiology, and endocrinology.

## Introduction

For the past several decades, iron oxide magnetic nanoparticles (IOMNPs) have attracted significant attention due to their broad potential use in environmental remediation (Wanna et al., 2016), data storage (Hyeon, 2003), adhesive hardening (Schmidt, 2005), and catalysis (Lu et al., 2004), as well as biotechnological applications such as magnetic hyperthermia (Nemati et al., 2016), cell labeling/separation (Lin et al., 2017), and magnetic resonance imaging (MRI) contrast enhancement (Li et al., 2016; Nakamura et al., 2017). One reason they are appropriate in the latter category of applications is their biocompatibility (Pankhurst et al., 2003). Their utility is defined by their propensity to be directed/guided with a static magnetic field in addition to their tendency to heat the local environment upon exposure to an alternating magnetic field (AMF). The physical mechanism behind this second characteristic is explained by linear response theory (LRT), which determines the heating efficacy of the IOMNPs based on their Néel and Brownian relaxation times (Dennis & Ivkov, 2013). Typically, superparamagnetic (SPM) response is desirable; the IOMNPs should exhibit no residual magnetization at zero applied magnetic field, and there should be no applied field necessary to return the magnetization to zero, also known as magnetic remanence and coercivity, respectively. This necessitates the employment of iron oxide only a few tens of nanometers in diameter (Lu et al., 2007).

On the contrary, poly(*N*-isopropylacrylamide) (PNIPAM) is regarded as the gold-standard, stimuli–responsive polymer (SRP). This is attributed to its biocompatibility and a lower critical solution temperature (LCST) near that of human body temperature. This LCST can easily be set above that of body temperature by way of changes in crosslinking density during polymerization (Schmaljohann, 2006). Like other SRPs (Sood et al., 2016), PNIPAM exhibits a nearly discontinuous volume phase transition (VPT) upon exposure to temperature or other stimuli like pH, electromagnetic fields, glucose, stress/strain, and ultrasound (Shibayama & Tanaka, 1993). This phase transition is explained in terms of the enthalpy/entropy dominance of PNIPAM’s Gibbs free energy. At temperatures below the LCST, the polymer is enthalpy dominated and water will hydrogen bind to its central amide section. Upon heating past the LCST, entropy dominates the Gibbs free energy and the isopropyl and acryl sections collapse on the amide section isolating it from surrounding water molecules (Schild, 1992). PNIPAM has proven to have appeal to researchers in a broad range of fields like tissue engineering (Healy et al., 2017), gene delivery (Zhang et al., 2016), waste water recovery (Ngang et al., 2017), sensors (Wang et al., 2017), and targeted drug delivery (Ahmad et al., 2016).

Conventional medical interventions typically involve exposing the entire patient to a biotherapeutic (e.g. doxorubicin, nucleic acids, etc.) when only a small fraction of the tissues require treatment. This is true in chemotherapy in which the patient often suffers from side effects, as well as gene therapy in which controversial genetic material is often used indiscriminately (Sun et al., 2008). What is wanted in these instances is a more precise administration of the biotherapeutic that could be achieved by the compositing of IOMNPs and PNIPAM. On one hand, the IOMNPs lend their honing capabilities to the application via attraction to a static magnetic field. They also enable environmental heating, of the medium in which they are dispersed, upon exposure to an AMF. On the other hand, PNIPAM is capable of carrying a multitudinous range of biotherapeutics and releasing them upon heating stimulus. Therefore, a targeted biotherapeutic delivery (TBD) device would be capable of carrying a biotherapeutic to a target tissue through remote guidance using a static field and triggered release of the biotherapeutic upon AMF exposure. Such devices promise to increase the efficacy of medical interventions by eliminating biotherapeutic waste, drastically reducing biotherapeutic exposure to otherwise healthy tissues, and actively targeting afflicted tissues (Martinez et al., 2012).

The incorporation of IOMNPs into PNIPAM nanogels has been achieved by several different methods. Often times emulsion polymerization is employed to synthesize the PNIPAM nanogels (Yuan & Wicks, 2007). This technique involves relatively long reaction times and employs the use of surfactants to ensure the nanogels have a narrow size distribution. More often than not the surfactants are not compatible with human biology and must be removed via dialysis adding expense and time to the process. A significantly different approach utilizes spray-dry synthesis of the PNIPAM nanogel (Byeon & Kim, 2012). Here, the resulting nanogels tend to require additional drying after synthesis to evaporate away excess water either in-flight or after collection. Furthermore, attempts to utilize nebulizers, rather than atomizers to achieve smaller initial droplets, are plagued with clogging of the equipment during synthesis (McLean et al., 1998).

Previously, the SRP concentration dependent LCST of PNIPAM as brought on by temperature increase due to AMF heating of co-dispersed IOMNPs was reported on (Denmark et al., 2015). While these SPM IOMNPs effectively raised the temperature of the co-solute PNIPAM bringing on its LCST, its mere presence had the consequence of reducing the LCST. This was attributed to an earlier onset of entropy dominance in the Gibbs free energy (Denmark et al., 2016). Herein, a unique method for the encapsulation of IOMNPs in a PNIPAM nanogel, via photpolymerization, coincident with in situ spectroscopic monitoring of that process is presented. Prior to encapsulation attempts, control of PNIPAM nanogel size is indicated by careful variation in the choice of monomer, crosslinker, and photoinitiator concentrations of the precursor solution. The separation of successfully embedded IOMNPs, within PNIPAM nanogels, from empty nanogels and bare IOMNP is demonstrated. Embedded IOMNPs are effective at heating their local environment upon AMF exposure as revealed by the corresponding heating curves. The results presented here will be invaluable to researchers endeavoring to advance in the realization of TBD devices.

## Materials and methods

### Materials

For the purposes of PNIPAM nanogel synthesis, the precursor solution was composed of solutes corresponding to the monomer, crosslinker, and photoinitiator. The monomer was *N*-isopropylacrylamide (NIPAM), the crosslinker was methylene bis-acrylamide (MBA), and the photoinitiator was Irgacure 2959. For all prepared solutions, the solvent was deionized water (DIW), which was also passed through a 220 nm porous cutoff filter for additional quality assurance. Both NIPAM and MBA were purchased from VWR (Radnor, PA) and the photoinitiator was purchased from Sigma Aldrich (St. Louis, MO). All solutes were used as received without further purification. In order to make any of the solutions discussed below the required amount of solute was weighed out and added to enough filtered DIW to yield the concentration indicated. After vigorous mechanical agitation, at least thirty 30 min of ultrasonic agitation was employed to ensure homogenous solutions. It should be noted that surfactants were not used in this work in order to make the product more compatible with biological applications like TBD.

### IOMNPs

The IOMNPs used in this work were characterized previously (Denmark et al., 2016). Briefly, their discreet size was measured by transmission electron microscopy (TEM) and found to be approximately 10 nm in diameter on average. Their magnetization as a function of applied magnetic field revealed a saturation magnetization of approximately 60 emu g^−1^ and no apparent coercivity, indicating they can be considered SPM. These IOMNPs were reported to be coated with PVP, for easy dispersal in water, by the manufacturer from which they were purchased (99.5% pure, US Research Nanomaterials, Inc., Houston, TX).

### *In situ* photopolymerization monitoring

**Figure 1.**
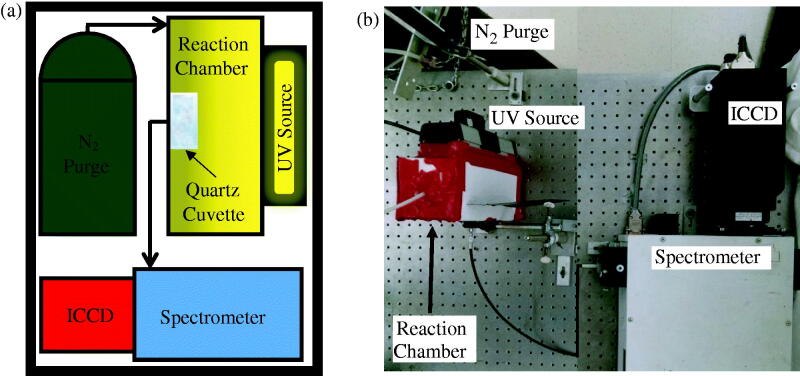
(a) Schematic and (b) photograph of experimental apparatus for photopolymerization synthesis of PNIPAM nanogels and *in-situ* monitoring.

The custom-built system that uniquely enabled the in situ monitoring of the ultraviolet (UV) source’s spectrum, while the photopolymerization synthesis of the PNIPAM nanogel was taking place, is shown in Figure 1. The source was mounted to the reaction chamber, which was sealed from the laboratory environment, so that its radiation could propagate into the chamber. Prior to the start of sample exposure, the chamber was purged with N_2_ long enough to purge it of O_2_ at least three times over. This was done to prevent the absorption of UV energy from the source before it could be used to power the synthesis. Similarly, a quartz cuvette was considered optimal for holding the precursor solution during UV photo-irradiation of the sample; plastic and glass cuvettes were found to absorb UV light during their characterization. Note, the synthesis requires no additional input of electrical energy to heat the sample, only what is required to power the source. Any light propagating through the precursor solution was transmitted by a fiber optic cable to a spectrometer and integrated charged coupled device for monitoring the spectrum of the source during photopolymerization. Again, for emphasis, any attenuation of the source as a result of the photoreaction was monitored by the same system carrying out the synthesis.

The UV source was a Hg gas filled discharge tube. In general, its spectrum was similar to that of standard Hg calibration tubes and well documented in NIST archives (data not shown). However, the selected source was specially coated on its glass tube to emit a significant UV peak at approximately 253.73 ± 0.03 nm and thought to be responsible for the cleavage of the photoinitiator molecule forming free radicals and ultimately leading to polymerization. The source was powered using a standard AC 60 Hz electrical power source. Since, positive or negative voltage powering of the source caused it to emit light, the emission frequency was approximately 120 Hz. Therefore, the source cycled once every 8 ms and was not continuous emission intensity. For this reason, it was decided to expose the ICCD detector to the source for a constant exposure time of 200 ms. In this way, any given exposure would represent the accumulation of nearly 25 individual pulses from the source, thus averaging out any error in intensity from each.

The spectrometer was a 1237 SpectraPro-500 manufactured by Acton Research Corporation (Greer, SC). Its focal length was 500 mm and was set to a resolution of 0.05 nm. Its wavelength operational range was from 185 nm to 850 nm: from UV to near infrared (NIR). The detector was a PI-MAX:512 UNIGEN Digital ICCD Camera System manufactured by Princeton Instruments Acton (Greer, SC). It was 512 × 512 pixels and specially coated for extra sensitivity in the UV. The equipment was programed with WinSpec software (Galactic Industries Corporation, Hamden, CT) to observe the source’s 546 nm peak for approximately 35 min. The reason for this is that, being the most prominent, the 546 nm peak was distinguishable amongst the surrounding noise as the source was attenuated during an experiment, as will be shown later.

### Turbidimetry

For the turbidimetry experiment described later on, a 25 mW He–Ne laser (CW Radiation, Inc., Mountain View, CA) of 633 nm wavelength was made to pass through the samples. The laser was detected on the other side of the sample with a cadmium–sulfide photodetector. That detector’s resistance was monitored with a Keithley 2100 multimeter.

### AMF heating

These experiments were carried out with an Ambrell EasyHeat LI heating system operating at applied magnetic fields of approximately 32.3 kA m^−1^ (405 Oe), 47.7 kA m^−1^ (600 Oe), and 59.1 kA m^−1^ (743 Oe). The corresponding operating frequencies at these field strengths were 313 kHz, 309 kHz, and 307 kHz. The temperature response of the samples was monitored with an optical temperature sensor (Photon Control) having an accuracy of ±0.05 °C. Note that initial experiments involved the exposure of a control sample, filtered DIW, to the fields described here so as to ascertain their heating response to the equipment. Despite being cooled with flowing water, the inductive heating coil, itself, generates heat that is unassociated with AMF heating. It is common practice to determine this heating response and subtract it from that recorded with samples actually containing IOMNPs (Simeonidis et al., 2013).

## Results and discussion

The nomenclature for the samples discussed in this work is provided in Table 1. Note that R1 refers to recipe 1 which indicates a monomer to crosslinker to photoinitiator ratio of 3:1:1 according to mass of the dissolved powder. R2 indicates a 10:1:1 ratio and R3 indicates a 1:1:1 ratio. The factor in front of a recipe name indicates to what extent that sample was diluted from its original stock solution. For example, 0.5R2 indicates the 10:1:1 sample was diluted to half its original concentration. These samples comprise the investigation into PNIPAM nanogel size tunability discussed later.

**Table 1.** Nomenclature of the precursor solution recipes investigated to demonstrate PNIPAM nanogel size control.

Sample	Dilution	NIPAM	MBA	Irgacure 2959
1.0R1	1.0	3	1	1
0.5R1	0.5	3	1	1
0.1R1	0.1	3	1	1
1.0R2	1.0	10	1	1
0.5R2	0.5	10	1	1
1.0R3	1.0	1	1	1

**Figure 2.**
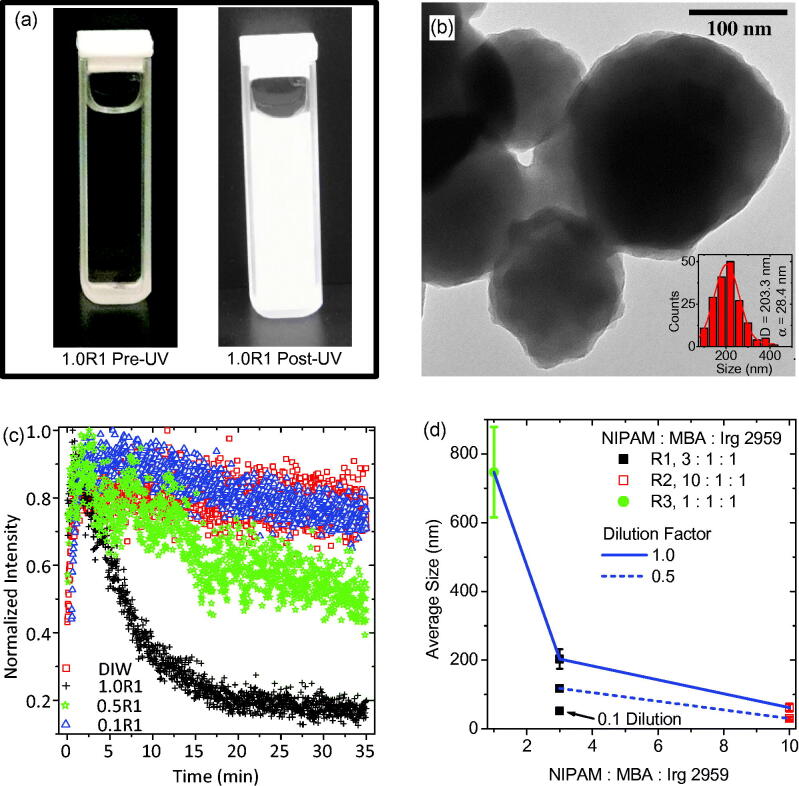
(a) A typical precursor solution, containing monomer, crosslinker, and photoinitiator dispersed in filtered DIW, before and after almost 35 min of UV exposure. (b) TEM of PNIPAM nanogels synthesized via UV exposure having an average size of approximately 200 nm as shown by the inset particle size distribution. (c) Intensity of the UV source as a function of elapsed exposure time quantifying the attenuation of the UV source. (d) Results of the precursor solution concentration study for demonstration of nanogel size control. This dilution study of average PNIPAM nanogel average size versus monomer to crosslinker ratio reveals that dilution leads to smaller nanogels and higher crosslinker content with respect to monomer leads to larger nanogels.

Upon mixing the monomer, crosslinker, and photoinitiator in filtered DIW, as described above, the solution appeared transparent and indistinguishable from water, as shown in Figure 2(a). After exposing the sample to the UV source for approximately 35 min, the front window of the cuvette (closest to the source) now exhibited a white film. The remainder of the solution took on a white, cloudy quality. This was the first indication of successful photopolymerization.

After an exposure, the sample was characterized using TEM for determination of PNIPAM nanogel size and morphology. As an example, the PNIPAM nanogels, synthesized using the 1.0R1 precursor solution, are shown in Figure 2(b). These nanogels are sphere-like and are on average approximately 200 nm in diameter. Note, the relatively light appearance of the nanogels; they appear to be empty.

The intensity of the UV source’s 546 nm peak is plotted against the duration of the exposure in Figure 2(c) for every dilution of precursor solution R1. Obviously, the source is being attenuated as the photopolymerization is taking place. The data presented in Figure 2(c) are a quantitative confirmation of the qualitative demonstration of source attenuation documented in Figure 2(a). The undiluted sample data (1.0R1) indicates that the synthesis is complete in only about 20–25 min. This is significantly faster than emulsion polymerization techniques, which can take several hours (Yuan & Wicks, 2007). Note, when precursor solution R1 is diluted to half of its original concentration the attenuation of the source is much less pronounced than in the undiluted sample. When the precursor solution is diluted to one tenth of its original concentration the attenuation of the bulb is indistinct from the control sample: plain filtered DIW. The reduction in intensity of the source seen by monitoring the filtered DIW appears to indicate the source is stabilizing over this time interval and could potentially be used to correct the other data sets for this effect. This obvious variation in intensity as a result in manipulation of the precursor solution’s concentration is reminiscent of turbidimetry studies for investigating the sizes of solutes dispersed in a solvent (Camerini-Otero & Day, 1978; Hall et al., 2016). In other words, Figure 2(c) seems to indicate that by diluting the precursor solution R1 different size PNIPAM nanogels have been achieved.

To verify PNIPAM nanogel size control, TEM was employed with all samples after UV exposure. The results of that study are plotted in Figure 2(d) in the form of a dilution factor study of average PNIPAM nanogel diameter as a function of monomer to crosslinker to photoinitiator ratio (horizontal axis). In this plot, dilutions are indicated according to: the nanogels resulting from undiluted precursor solutions are connected by a solid blue line, those diluted by half are connected by a dashed blue line, and those diluted by a tenth are indicated by an arrow and label. In general, the diameter of the nanogels was made to range from as small as almost 40 nm to as large as almost 750 nm. In terms of the precursor solution dilution, increased potency led to larger nanogels. In terms of monomer to crosslinker ratios, the more concentrated the crosslinker was the larger the nanogels were. This is consistent with the findings of other researchers (Neyret & Vincent, 1997; Duracher et al., 2000). Ultimately, the largest PNIPAM nanogel recipe (1.0R3) was considered optimal for the IOMNP encapsulation study discussed next, because larger nanogels would conceivably have more carrying capacity making encapsulation more likely.

**Figure 3.**
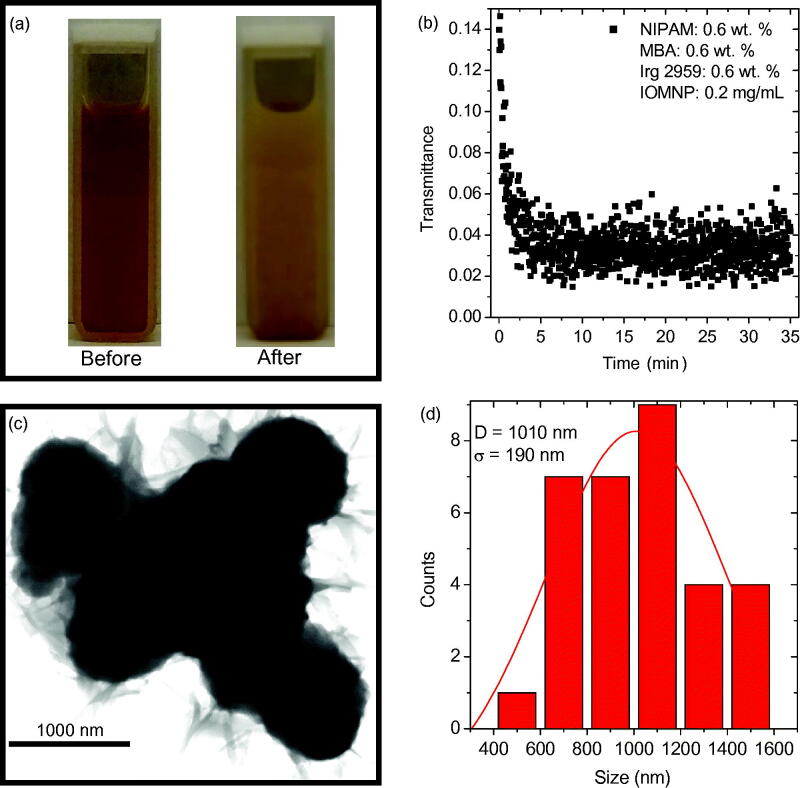
(a) Photographs of the precursor solution 1.0R3 with 0.2 mg mL^−1^ IOMNPs dispersed therein before and after being exposed to the UV source for about 35 min. (b) The transmittance of the UV source through the sample photographed in (a) as a function of elapsed exposure time. (c) TEM of the PNIPAM nanogels synthesized, in the presence of IOMNPs, via UV exposure. (d) A particle size distribution of the nanogels shown in (c) revealing an average size of approximately 1000 nm.

For encapsulation, the precursor solution 1.0R3 had enough IOMNPs added to it to bring their concentration to 0.2 mg mL^−1^. This caused the formerly transparent sample (see Figure 2(a)) to take on a reddish, brown quality, as seen in Figure 3(a), which can be attributed to the presence of the IOMNPs. After the sample was exposed to the UV source for about 35 min, it again exhibits a film on the cuvette window closest to the source, also shown in Figure 3(a). These photographs qualitatively document the cause of the UV source’s attenuation as synthesis progresses.

The transmittance of the UV source’s 546 nm peak as a function of exposure duration is shown in Figure 3(b). These data represent the attenuation of the UV source quantitatively. With IOMNPs added to the sample the synthesis was complete after only 5–10 min. Note the initial low transmittance of the sample, at the beginning of the exposure, which can be attributed to the presence of the IOMNPs.

An aliquot of this sample was prepared for TEM imaging and the resulting nanogels are shown in Figure 3(c). Note that these nanogels are apparently much darker than those shown in Figure 2(b). In other words, it appears that IOMNPs could be within the nanogels. Furthermore, the particle size distribution of these nanogels reveals an average size of nearly 1000 nm, significantly larger than those synthesized in the absence of IOMNPs. If a perfectly spherical nanogel is assumed, the nanogels synthesized in the presence of IOMNPs have an approximate volume of 0.52 cubic microns, while those synthesized without IOMNPs have an approximate volume of 0.22 cubic microns. This means those synthesized in the presence of IOMNPs have over twice the volume as those made without IOMNPs dispersed in the solution. This certainly seems to suggest that the PNIPAM nanogels shown in Figure 3(c) appear to encapsulate IOMNPs.

Granted, not all species within the post-UV exposed sample are PNIPAM embedded IOMNPs. Certainly, some IOMNPs will remain non-embedded and so will be referred to as bare. This species, being most dense, will be most susceptible to gravitational forces. At the same time, some empty PNIPAM nanogels can be expected. This species is least dense and so least susceptible to gravity. Note that the embedded IOMNPs are susceptible to gravitational forces, while the empty PNIPAM nanogels are not. As further evidence of successful IOMNP encapsulation, their unique magnetic quality was employed. Two identical aliquots of the post UV exposed sample were prepared and vigorously agitated to ensure they were homogenously dispersed throughout. The control sample was set on the laboratory counter over a 16-h period without being disturbed except for gentle testing as described further down. This control sample had only the gravitational force to act on its contents. The experimental sample was also left alone on the laboratory counter for the same period of time, but it had a small neodymium magnet placed adjacent to one of the walls of its container, far from the bottom so as not to disturb any bare IOMNPs that settled there. Now that each sample was probed with a He–Ne laser both immediately after being prepared and again 16 h after being allowed to sit with their respective forces acting on them. Similarly, a sample of filtered DIW was probed so that transmittance could be determined. The initial transmittances of both the control and the experimental sample were similar, as expected. However, after 16 h, the sample with the magnet in proximity to it exhibited significantly higher transmittance than the sample that only had gravity working on its contents. This is further evidence of at least some degree of successful IOMNP encapsulation within the PNIPAM nanogel and suggests separation from the other species is possible. These results are summarized in Supplemental Table 1.

**Figure 4.**
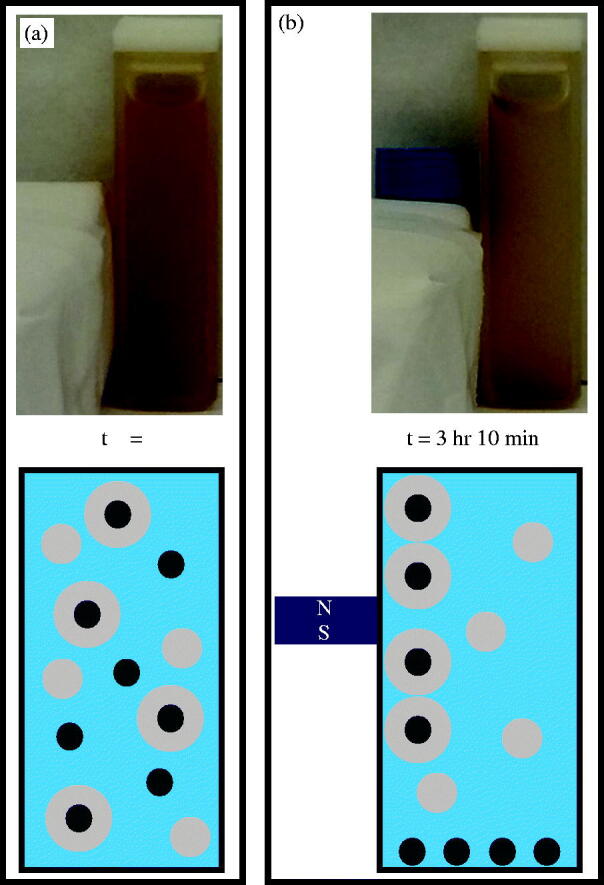
(a) The sample was agitated after UV exposure, thereby suspending bare IOMNPs, empty nanogels, and PNIPAM embedded IOMNPs in the water column. (b) Bare IOMNPs settle relatively fast under the influence of gravity. A static magnetic field can be made to isolate embedded IOMNPs, from the empty nanogels and bare IOMNPs, along the cuvette wall.

Separation of the successfully embedded IOMNPs from the other species is presented next. This separation was achieved in a facile manner by employing gravity and a magnet on the post UV exposed sample. First the sample was allowed to sit undisturbed for nearly two hours so that gravity could effectively remove the bare IOMNPs from the water column. After this, a small neodymium magnet was located adjacent to a wall of the cuvette, relatively far from its bottom so as not to disturb the bare IOMNPs settled there. A little over 3 h later, the separation was achieved as illustrated photographically and schematically in Figure 4. Note that at the beginning of the separation process the sample had been vigorously agitated so as to achieve homogeneity. As shown in the photograph and schematic of Figure 4(a), this distributes all three species equally throughout the water column. After separation, as shown in Figure 4(b), two distinct regions of reddish, brown are photographically documented at the cuvette bottom and wall nearest the magnet. These can be attributed to the relatively strong magnetic field here as magnetic field lines converge on the poles. The schematic of Figure 4(b) depicts how the successfully embedded IOMNPs have been isolated to the wall of the cuvette. This, then, allows the embedded IOMNPs to be washed and permanently isolated from the unwanted species. Note the sample’s separation behavior, documented here in Figure 4, was repeatable over an eight month period of time suggesting the synthesis is stable over at least that long, provided it is refrigerated at about 4 °C when stored.

**Figure 5.**
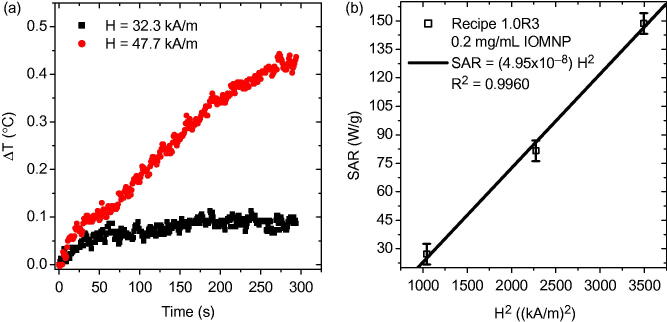
(a) The AMF heating curves of the sample comprised of precursor solution 1.0R3 and IOMNPs after being exposed to the UV source. (b) The SAR as a function of the square of the applied magnetic field amplitude.

Now that the separated sample was assessed for AMF heating response at several different field strengths. The heating response of the PNIPAM embedded IOMNPs, at 32.3 kA m^−1^ and 47.7 kA m^−1^, is given in Figure 5(a). Obviously, by varying the strength of the AMF the heating response can be controlled. In particular, stronger applied fields (47.7 kA m^−1^) induce elevated temperature increase (∼0.4 °C) as compared with weaker applied fields (32.3 kA m^−1^) which induce less temperature increase (∼0.1 °C) for a given trial duration. Heating rates, however, can only inform on the heating efficacy of the entire sample. In order to comment on the heating efficacy of the individual IOMNPs the specific absorption rate (SAR) must be determined according to

(1) $$\text{SAR}\;\text{=}\frac{{\text{c}}_{\text{sol}}\;m_{\text{sol}}}{m_{\text{IOMNP}}}\;\frac{\text{Δ}T}{\text{Δ}t}$$

In Equation (1), the subscript sol indicates that the product in the numerator is the sum of the three terms corresponding to the components of the solution: the IOMNPs, the PNIPAM, and the filtered DIW. The quantity c indicates the specific heat of a particular species and *m* is its mass. The quantity Δ*T* is the change in temperature for a given time interval, Δ*t*. In Equation (1), since the heating rate of a sample is normalized by the mass of the IOMNPs, *m*_IOMNP_, it is independent of that quantity and can, therefore, comment on their individual heating efficacy. According to linear response theory (LRT) SAR is proportional to the square of the applied magnetic field strength, H^2^ (Rosensweig, 2002). When the SAR is plotted against H^2^ the result is indeed a linear relation, as shown in Figure 5(b). It was previously reported that when IOMNPs are dispersed by themselves in DIW, with no PNIPAM present, at a concentration of 1 mg mL^−1^ the SAR was approximately 220 W g^−1^ (Denmark et al., 2016). That data corresponded to a field strength of 59.1 kA m^−1^. However, here, when IOMNPs were embedded in PNIPAM the SAR was approximately 150 W g^−1^. This reduction in SAR can be attributed to a confining action of the PNIPAM nanogel matrix on the IOMNPs thus reducing their heating efficacy.

When IOMNPs are not coated with surfactants they have a strong tendency to cluster together as a result of dipolar particle interactions. However, in the case of the IOMNPs that were encapsulated within PNIPAM, described above, interactions are weak. It follows that IOMNP/PNIPAM nanogels will disperse better in the solvent. Through multiple hyperthermia experiments, the SAR value was the same, meaning the PNIPAM nanogels containing IOMNPs are homogeneously dispersed.

Through the use of Equation (1), estimation of the amount of encapsulated IOMNPs within the PNIPAM nanogels is possible. Since bare IOMNPs exhibited a SAR value of 220 W g^−1^ and the IOMNP/PNIPAM nanogels had a SAR value of 150 W g^−1^, this suggests that approximately 70% of the initial amount of IOMNPs were successfully encapsulated.

## Conclusions

The work presented here furthers the field of TBD. Herein, a unique system was presented that not only allows for photopolymerization synthesis of PNIPAM nanogels encapsulating IOMNPs, but is also capable of monitoring that synthesis in situ through recording the attenuation of the source’s spectrum as the photopolymerization progresses. By employing a photoreaction, the synthesis was faster, energetically cheaper, and safer for use in biological applications as compared to more traditional techniques like emulsion polymerization. Through tuning the initial concentration of the precursor solution, the final PNIPAM nanogel size could be dialed in. Finally, effective separation of the PNIPAM embedded IOMNPs from the unreacted species was demonstrated in a facile manner by employing gravitational and magnetic forces. The results presented here will be of interest to those researchers endeavoring to develop their own TBD devices.

## References

[CIT0001] Ahmad N, Ahmad I, Umar S, et al. (2016). PNIPAM nanoparticles for targeted and enhanced nose-to-brain delivery of curcuminoids: UPLC/ESI-Q-ToF-MS/MS-based pharmacokinetics and pharmacodynamic evaluation in cerebral ischemia model. Drug Deliv 23:2095–114. 25237726 10.3109/10717544.2014.941076

[CIT0002] Byeon JH, Kim J-W. (2012). Aerosol fabrication of thermosensitive nanogels and in situ hybridization with iron nanoparticles. Appl Phys Lett 101:023117.

[CIT0003] Camerini-Otero RD, Day LA. (1978). The wavelength dependence of the turbidity of solutions of macromolecules. Biopolymers 17:2241–9.

[CIT0004] Denmark DJ, Bradley J, Mukherjee D, et al. (2016). Remote triggering of thermoresponsive PNIPAM by iron oxide nanoparticles. RSC Adv 6:5641–52.

[CIT0005] Denmark DJ, Mukherjee D, Bradley J, et al. (2015). Systematic study on the remote triggering of thermoresponsive hydrogels using RF heating of Fe3O4 nanoparticles. Mater Res Soc Symp Proc 1718:1718-b04-30.

[CIT0006] Dennis C, Ivkov R. (2013). Physics of heat generation using magnetic nanoparticles for hyperthermia. Int J Hyperthermia 29:715–29. 24131317 10.3109/02656736.2013.836758

[CIT0007] Duracher D, Elaissari A, Pichot C. (2000). Effect of a cross-linking agent on the synthesis and colloidal properties of poly(*N*-isopropylacrylamide) microgel latexes. Macromol Symp 150:305–11.

[CIT0008] Hall D, Zhao R, Dehlsen I, et al. (2016). Protein aggregate turbidity: simulation of turbidity profiles for mixed-aggregation reactions. Anal Biochem 498:78–94. 26763936 10.1016/j.ab.2015.11.021

[CIT0009] Healy D, Nash M, Gorelov A, et al. (2017). Fabrication and application of photocrosslinked nanometer-scale, physically adsorbed films for tissue culture regeneration. Macromol Biosci 17:1600175. 10.1002/mabi.201600175 27584800

[CIT0010] Hyeon T. (2003). Chemical synthesis of magnetic nanoparticles. Chem Commun 2003:927–34. 10.1039/b207789b 12744306

[CIT0011] Li L, Gao F, Jiang W, et al. (2016). Folic acid-conjugated superparamagnetic iron oxide nanoparticles for tumor-targeting MR imaging. Drug Deliv 23:1726–33. 25715808 10.3109/10717544.2015.1006404

[CIT0012] Lin R, Li Y, MacDonald T, et al. (2017). Improving sensitivity and specificity of capturing and detecting targeted cancer cells with anti-biofouling polymer coated magnetic iron oxide nanoparticles. Colloids Surf B Biointerfaces 150:261–70. 28029547 10.1016/j.colsurfb.2016.10.026 PMC5253252

[CIT0013] Lu A-H, Salabas EL, Schüth F. (2007). Magnetic nanoparticles: synthesis, protection, functionalization, and application. Angew Chem Int Ed Engl 46:1222–44. 17278160 10.1002/anie.200602866

[CIT0014] Lu A-H, Schmidt W, Matoussevitch N, et al. (2004). Nanoengineering of a magnetically separable hydrogenation catalyst. Angew Chem Int Ed Engl 43:4303–6. 15368378 10.1002/anie.200454222

[CIT0015] Martinez J, Brown B, Quattrocchi N, et al. (2012). Multifunctional to multistage delivery systems: the evolution of nanoparticles for biomedical applications. Chin Sci Bull 57:3961–71. 24587616 10.1007/s11434-012-5387-5 PMC3938208

[CIT0016] McLean JA, Minnich MG, Iacone LA, et al. (1998). Nebulizer diagnostics: fundamental parameters, challenges, and techniques on the horizon. J Anal At Spectrom 13:829–42.

[CIT0017] Nakamura M, Hayashi K, Kubo H, et al. (2017). Relaxometric property of organosilica nanoparticles internally functionalized with iron oxide and fluorescent dye for multimodal imaging. J Colloid Interface Sci 492:127–35. 28086116 10.1016/j.jcis.2017.01.004

[CIT0018] Nemati Z, Alonso J, Martinez L, et al. (2016). Enhanced magnetic hyperthermia in iron oxide nano-octopods: size and anisotropy effects. J Phys Chem C 120:8370–9.

[CIT0019] Neyret S, Vincent B. (1997). The properties of polyampholyte microgel particles prepared by microemulsion polymerization. Polymer 38:6129–34.

[CIT0020] Ngang H, Ahmad A, Low S, Ooi B. (2017). Preparation of thermoresponsive PVDF/SiO2-PNIPAM mixed matrix membrane for saline oil emulsion separation and its cleaning efficiency. Desalination 408:1–12.

[CIT0021] Pankhurst QA, Connolly J, Jones SK, Dobson J. (2003). Applications of magnetic nanoparticles in biomedicine. J Phys D: Appl Phys 36:R167–81.

[CIT0022] Rosensweig RE. (2002). Heating magnetic fluid with alternating magnetic field. J Magn Magn Mater 252:3703742002.

[CIT0023] Schild H. (1992). Poly(*N*-isopropylacrylamide): experiment, theory, and application. Prog Polym Sci 17:163–249.

[CIT0024] Schmaljohann D. (2006). Thermo- and pH-responsive polymers in drug delivery. Adv Drug Deliv Rev 58:1655–70. 17125884 10.1016/j.addr.2006.09.020

[CIT0025] Schmidt AM. (2005). Induction heating of novel thermoresponsive ferrofluids. Magn Magn Mater 289:5–8.

[CIT0026] Shibayama M, Tanaka T. (1993). Volume phase transition and related phenomena of polymer gels. Adv Polym Sci 109:1–62.

[CIT0027] Simeonidis K, Martinez-Boubeta C, Balcells L, et al. (2013). Fe-based nanoparticles as tunable magnetic particle hyperthermia agents. J Appl Phys 114:103904.

[CIT0028] Sood N, Bhardwaj A, Mehta S, Mehta A. (2016). Stimuli–responsive hydrogels in drug delivery and tissue engineering. Drug Deliv 23:748–70. 10.3109/10717544.2014.940091 25045782

[CIT0029] Sun C, Lee J, Zhang M. (2008). Magnetic nanoparticles in MR imaging and drug delivery. Adv Drug Deliv Rev 60:1252–65. 18558452 10.1016/j.addr.2008.03.018 PMC2702670

[CIT0030] Wang T, Yu Y, Chen D, et al. (2017). Naked eye plasmonic indicator with multi-responsive polymer brush as signal transducer and amplifier. Nanoscale 9:1925–33. 28098296 10.1039/c6nr09631j

[CIT0031] Wanna Y, Chindaduang A, Tumcharern G, et al. (2016). Efficiency of SPIONs functionalized with polyethylene glycol bis (amine) for heavy metal removal. J Magn Magn Mater 414:32–7.

[CIT0032] Yuan B, Wicks DA. (2007). Thermotropic color changing nanoparticles prepared by encapsulating blue polystyrene particles with a poly-*N*-isopropylacrylamide gel. J Appl Polym Sci 105:446–52.

[CIT0033] Zhang H, Li Q, Zhang Y, et al. (2016). A nanogel with passive targeting function and adjustable polyplex surface properties for efficient anti-tumor gene therapy. RSC Adv 6:84445–56.

